# Notes and News

**Published:** 1894-11-10

**Authors:** 


### Notes and News.

Collected and Communicated.

The Lord Mayor, the Lady Mayoress, the Sheriffs of London and Middlesex, and other City dignitaries paid a visit to the London Temperance Hospital last week. The Lord Mayor expressed himself as most favourably impressed by his visit, and indeed those would be difficult to please to whom the bright wards of this hospital did not appeal.

Mr. Sydney Holland, that most active of chairmen, is issuing an attractive and effective appeal on behalf of the Poplar Hospital for Accidents. Attached to the appeal is a drawing of the hospital as now completed, which shows the great improvements that have been effected as seen from the outside. The new hospital cost £30,000, the whole of which sum has been paid, and further we learn that the hospital has never been in debt. Such careful financing of an institution situated in the heart of a poverty-stricken neighbourhood ought to inspire the richer citizens to come forward and help to the best means in their power, as subscriptions and donations are urgently needed.

The proceedings at the meetings of the Bedford Board of Guardians on several recent occasions should attract and fix the attention of every ratepayer in Bedford. No unprejudiced person who has any knowledge of the true inwardness of workhouse administration can doubt that the interests of the poor demand a thorough reorganisation of the internal management of the Bedford Workhouse. We have not space to go into details, but two lady visitors in a letter to the *Bedfordshire Times* state the case in a nutshell. The present Board of Guardians instead of instituting inquiry have simply burked, and continue to burke the whole question. They write, "What we and a great many others in Bedford want"—and what, we may add, the public at large will insist upon securing—"is not a solid plumper for the good master, but proper inspection, inquiry, and supervision of the wards of the workhouse." We have confidence that the ratepayers of Bedford will so alter the personnel of the present Board of Guardians at the ensuing elections as to secure the speedy application of an effectual remedy to the undoubted evils which at present exist to the injury of the reputation of the town and county.

Paris may well be proud of its record of seven weeks' immunity from small-pox cases. This excellent state of affairs M. Hervieux attributes to the fact that revaccination is most rigorously carried out.

At the last meeting of the Metropolitan Hospital Saturday Fund Mr. R. Acland, the chairman, proposed the formation of a "premises fund," owing to the inadequacy of the present accommodation for the increasing work of the fund.

Lord Sandhurst opened a hospital at Waltham Cross last week. The Royal Gunpowder Factory, which is situated in the neighbourhood, has already caused sufficient accidents to render an institution for the reception of sufferers to be necessary. The new hospital contains 12 beds, and is placed in the charge of a thoroughly qualified nurse; the factory surgeon will act as medical officer.

The action taken in Glasgow in establishing a Hospital Sunday Fund collection is a new departure in Scotland. Glasgow has taken the matter warmly up; it realises that prompt action is necessary to secure support for their hospitals, and that a Sunday Fund will tend to this end. The three great infirmaries, the Royal, the Western, and the Victoria show together an adverse balance of £17,000 on their accounts. Three hundred congregations and 200 Sabbath schools have been and are still making collections. Up to the time of publishing an average of £11 13s. 9d. per collection has been received.

The desirability of large orphanages is very much questioned nowadays, both on the point of economy and as to the actual advantages accruing to the children. Modern requirements are constantly forcing expensive additions and alterations on all large institutions. At the Mason Orphanage, Birmingham, for instance, it has now been considered necessary to erect an isolation building in connection with the home. Epidemics are common to all large assemblages of children, and the expense to the ratepayers or voluntary supporters of institutions containing these communities is very large. Where children are placed in small detachments, or singly in the homes of villagers, the expenses are very much better distributed, and when future need of provision arises to provide further accommodation for orphans, it is to be hoped the experience of the past will be carefully studied.

The foundation-stone of the first permanent home on the farm colony for epileptics at Chalfont St. Peters, in Buckhamshire, will be laid on the 14th inst. by Mr. Passmore Edwards. The colony was opened on August 1st, a temporary iron building having been erected for the accommodation of eighteen epileptics. The colonists have been engaged chiefly in outdoor work upon the land, and the experiment, it is stated, has so far been attended with the most encouraging results. Though the society have acquired, through the generosity of Mr. Passmore Edwards, enough land to form the site of a colony for some hundreds of inhabitants, their present funds will be almost exhausted in the erection of a permanent building for about twenty men, leaving women and children totally unprovided for. The Committee of the National Society for Employment of Epileptics most earnestly appeal for help to enable them to extend their work, the need for which is undoubted.

